# Veterinary Medical Society, University of Pennsylvania

**Published:** 1900-01

**Authors:** Harry E. Bender

**Affiliations:** Secretary


Secretary.

### VETERINARY MEDICAL SOCIETY, UNIVERSITY OF PENNSYLVANIA.

THE meeting was called to order at 8 o'clock P.M., November 24, 1899. Mr. Tallman, '00, was appointed critic. It was moved, seconded, and carried to dispense with the regular order of business.

Mr. Young, '00, then read an excellently prepared paper on "Irregularities of the Dental Apparatus."

Dr. Pearson, Dean of the Veterinary Department, then addressed the Society on "Horse Breeding." He gave an elegant address, and showed the members photographs of the various breeds of horses which are important to students of veterinary medicine. A vote of thanks was extended to Dr. Pearson, and also to Mr. Young.

Discussion of the banquet was then taken up. It was moved, seconded, and carried that the Chair appoint a committee of three to arrange for the banquet.

The Chair then appointed the following committee: Messrs. Cornman, '00, Carlisle, '01, and McCloskey, '02.

Next in order was the Treasurer's report. This was his first report, and in it he stated the financial condition of the Society, which was very encouraging.

The critic's report was very favorable.

Adjourned at 9.30 P.M.

*December* 18, 1899.

The meeting was called to order at 7.15 o'clock P.M., on Friday, December 8th. Mr. Cornman, '00, was appointed critic.

It was moved, seconded, and carried to renew our subscription for the *Pennsylvanian*.

The Librarian was authorized to look into the matter of having the papers brought into the reading-room, and also to report what was being done in regard to printing the By-laws.

The Supper Committee reported that they had selected the Hotel Aberdeen for the banquet.

It was moved, seconded, and carried that the banquet be held December 16th, at 9 o'clock P.M. It was also moved, seconded, and carried that the Secretary invite by letter the three clinical professors and also the resident surgeon.

Mr. Van Sant was then proposed and elected to membership.

The critic's report was favorable.

Adjourned at 8.30 P.M.

The third annual banquet of the Society was held on the evening of the 16th of December at the Hotel Aberdeen. There was a large attendance, and the evening was very pleasantly spent. At a seasonable hour an elegant supper was spread, which was greatly enjoyed by all.

This affair has been enrolled in the archives of the Association as the greatest social function in the history of its existence, and to use a by-word of our alma mater, "It is important to the veterinarian."

The affair was voted by all present to be a great success.

HARRY E. BENDER,
Secretary.

